# Recombinant chicken interleukin-7 as a potent adjuvant increases the immunogenicity and protection of inactivated infectious bursal disease vaccine

**DOI:** 10.1186/s13567-017-0497-3

**Published:** 2018-02-01

**Authors:** Dan Cui, Jianlou Zhang, Yuzhu Zuo, Shanshan Huo, Yonghong Zhang, Liyue Wang, Xiujin Li, Fei Zhong

**Affiliations:** 1 Laboratory of Molecular Virology and Immunology, College of Veterinary Medicine/College of Animal Science and Technology, Agricultural University of Hebei, Baoding, 071000 Hebei China; 2 Hebei Engineering and Technology Research Center of Veterinary Biotechnology, Baoding, 071000 Hebei China; 3 Department of Biotechnology, College of Environmental and Chemical Engineering, Yanshan University, Qinhuangdao, 066004 Hebei China

## Abstract

Our previous work showed that a plasmid-based chicken interleukin-7 (chIL-7) gene expression vector possessed potent adjuvant activity for a VP2 DNA vaccine against chicken infectious bursal disease virus (IBDV). Whether recombinant chIL-7 prepared in procaryotic expression system has the adjuvant activity for inactivated IBDV vaccine remains unknown. Here, we prepared recombinant chIL-7 using an *E. coli* expression system and analyzed its adjuvant activity for the inactivated IBDV vaccine. The results show that the recombinant chIL-7 was successfully prepared in *E. coli* using the pET20b vector, which possessed biological activity to stimulate mouse B lymphocyte proliferation. Co-administration of the chIL-7 with inactivated IBDV vaccine significantly increased specific serum antibody titers against IBDV, enhanced lymphocyte proliferation and IFN-γ and IL-4 productions, and increased protection against virulent IBDV infection.

## Introduction

Infectious bursal disease (IBD), an acute and highly contagious chicken disease, is caused by infection with IBD virus (IBDV) and characterized by virus-induced immunosuppression in young chickens mainly via destruction of antibody-producing B cells in the bursa of Fabricius [1, 2]. The virus replicates rapidly in developing B cells causing the destruction of the precursors of antibody-producing B cells in the bursa, resulting in immunosuppression leading to vaccination failure and susceptibility to other microbial infections [3]. Therefore, IBD is considered one of the most important viral diseases threatening the poultry industry worldwide [4].

So far, IBD has not been completely controlled although vaccination programs have been extensively implemented worldwide using live attenuated or inactivated IBDV vaccines. Outbreaks of IBD still occur [5], and are often accompanied by the emergence of highly virulent and variant strains, probably due to selection pressure from the administration of live attenuated IBDV vaccine [6]. In addition, the live IBDV vaccine has often caused chicken immunosuppression and sub-clinical infection [7], whereas the inactivated IBDV vaccine has not completely protected chickens from virulent IBDV attack [8]. Therefore, development of potent adjuvants may be required to enhance the immunogenicity of inactivated IBDV vaccines.

In recent years, many adjuvants for inactivated IBDV vaccine have been investigated. Besides conventional adjuvants (such as alum, liposomes, microparticles and oil/water emulsions), biological adjuvants have been investigated, mainly involving recombinant polypeptides [9], amino acids [10], active components of Chinese traditional medicine [11] and plasmid-based cytokine genes, including IL-2 [12, 13], IL-6 [14], IL-7 [15, 16], IL-18 [17] and IFN-γ [18, 19] genes, to enhance IBDV VP2 DNA vaccine immunogenicity. However, recombinant proteins as the biological adjuvants (recombinant cytokines or other recombinant proteins) for inactivated IBDV vaccine have only received limited attention. Only recombinant chicken IL-12 protein [20], recombinant chicken thymic hormone [21] and recombinant polypeptide [9] have been investigated.

IL-7 is a pleiotropic cytokine produced by bone marrow and thymic stromal cells, which was originally discovered as a pre-B cell growth factor [22] playing a crucial role in initiating and maintaining activities of the immune and hematopoietic systems [23, 24]. IL-7 not only stimulates B cell differentiation, proliferation, maturation and maintenance [25, 26], but also stimulates T cell development, proliferation and homeostatic regulation [27, 28]. Due to its potent immunity-stimulating property, IL-7 has been used in humans to treat certain immunosuppressive diseases [29, 30] and to enhance vaccine immunogenicity as a biological adjuvant [31, 32].

Recently we prepared recombinant chIL-7 from a eukaryotic expression system and demonstrated that it possessed antiviral activity against IBDV [15, 16]. We also showed that a plasmid-based chIL-7 gene vector could enhance the immunogenicity of IBDV VP2 DNA vaccine [15, 16]. However, whether recombinant chIL-7 could function as an adjuvant to enhance inactivated IBDV vaccine efficiency was not established. Like other species in IL-7 structure, chIL-7 is a glycosylated protein with four potential *N*-glycosylated sites in chIL-7 protein. Whether *E coli* system-expressed unglycosylated chIL-7 retains biological activity and possesses adjuvant activity for IBDV vaccines is unknown. Therefore, in this study, we constructed a chIL-7 prokaryotic vector, expressed it in the BL21 *E. coli* expression system and analyzed its ability to stimulate B cell production and its adjuvant activity.

## Materials and methods

### Plasmids, cells, viruses and chickens

The pET20b(+) plasmid, a prokaryotic expression vector, was purchased from Novagen. pcDNA-chIL-7/MH plasmid containing the full-length chicken IL-7 gene was previously constructed [15, 16]. Mouse immature B lymphocytes (2E8 cells) for IL-7 activity assay were from ATCC (Manassas, VA). A virulent IBDV strain (Harbin-1 strain) [33] was kindly provided by Dr Zandong Li, China Agricultural University, and propagated in 10-day-old specific-pathogen-free (SPF) embryonated chicken eggs from SPF chickens (Jinan Sais Poultry Co.) maintained in an isolator in an environmentally controlled room with a 12/12 h light/dark cycle. Animal experiments were approved by the Animal Ethics Committee of the Agricultural University of Hebei.

### Preparation of inactivated IBDV vaccine

A virulent strain of IBDV (Harbin-1 strain) was used for construction of inactivated IBDV vaccine. The viruses were propagated in SPF embryonated chicken eggs for 5 days at 37 °C, collected from allantoic fluid, titered by 50% embryo lethal doses (ELD_50_) as calculated by the Reed–Muench method and inactivated with 0.4% formaldehyde for 24 h, and then suspended in PBS (pH 7.2). After confirming that the virus was completely inactivated, they were emulsified in 2% aluminum stearate to produce the inactivated IBDV vaccines.

### Proteins and antibodies

Recombinant IBDV VP2 protein was prepared in our laboratory as described previously [15, 16]. Mouse anti-His antibody and horseradish peroxidase (HRP)-conjugated goat anti-mouse IgG (IgG-HRP) (sc-2031) were purchased from Santa Cruz Biotechnology.

### Construction of chIL-7 expression vector

To construct the chIL-7 prokaryotic expression vector, the signal peptide-deleted chIL-7 gene was amplified from pcDNA-chIL-7/MH plasmid by PCR using the following primers: chIL7-Fnsp: CATGCCATGG(*Nco*I)ATTCTAGCTGTACAATGGGAAATAAAAC; chIL7-Rns: CCCGCTCG AG (*Xho*I)ACACCTTGAAATTATTTTTTCAAATTTATTC. The chIL-7 gene was inserted into the pET20b(+) vector by *Nco* I and *Xho* I sites to generate chIL-7 prokaryotic expression vector pET20b-chIL-7/H. the chIL-7 gene was fused in the vector pET20b-chIL-7/H with the *pelB* signal peptide at the N-terminus for secretory expression (potential periplasmic localization) and a His-tag at the C-terminus for chIL-7 detection using Western blot and purification with Ni–NTA agarose beads.

### Expression in *E. coli*

*Escherichia coli* BL21 (DE3) competent cells were transformed with the pET20b-chIL-7/H vector, and positive transformed *E. coli* were cultured overnight in 5 mL Luria–Bertani (LB) medium containing 100 μg/mL ampicillin at 37 °C. Cultures were then added to fresh 100 mL LB media in 500 mL flasks and allowed to continue to grow at 30 °C with shaking (200 rpm). When the optical density at 600 nm (*OD*_600_) of the culture reached 0.6, isopropyl-β-d-thiogalactopyranoside (IPTG) was added to a final concentration of 1 mM to induce chIL-7 expression. The *E. coli* were cultured for four additional hours and then harvested by centrifugation at 8000 × *g* for 5 min at 4 °C. Bacterial pellets were resuspended in 30 mL suspension buffer (30 mM Tris–HCl, 20% sucrose, pH8) and 60 μL 0.5 mol/L EDTA (pH8) was added into the suspension (final concentration 1 mM) and stirred for 10 min at room temperature. The bacteria were collected by centrifugation at 4 °C for 10 min at 8000 *g*, resuspended in 30 mL ice-cold 5 mM MgSO_4_, and stirred in an ice bath for 10 min, resulting in the release of periplasmic proteins including chIL-7 into the medium. Following centrifugation at 10 000 *g* for 10 min at 4 °C, the supernatant containing recombinant chIL-7 was collected for purification.

### Purification of recombinant chIL-7

The final supernatants were mixed with an Ni–NTA-agarose bead slurry (20:1 v/v) and incubated overnight at 4 °C with shaking for immobilization of His-tag fused chIL-7. After precipitation, the beads were washed 4× with washing buffer (20 mM Tris, 500 mM NaCl, 10 mM imidazole pH 8.0). Bound chIL-7 protein was eluted with elution buffer (20 mM Tris, 500 mM NaCl, 250 mM imidazole pH 8.0) and dialysed against PBS (pH 8.0) to eliminate imidazole. Protein concentration was determined using the Bio-Rad protein assay kit (Bio-Rad) developed based on the method of Bradford [34].

### SDS-PAGE and Western blot

The recombinant chIL-7 preparations were subjected to 10% SDS-PAGE and the protein bands were either stained with Coomassie Brilliant Blue G250 for protein purity analysis or transferred onto a nitrocellulose membrane (Hybond-C, Amersham Pharmacia) for Western blot detection. The transferred membrane was blocked with 5% non-fat milk in PBST for 2 h at room temperature before incubation with mouse anti-His antibody (1:1000 dilution) for 2 h at 37 °C, followed by goat anti-mouse IgG-HRP (1:1000 dilution) for 1.5 h at 37 °C. Bands were visualized with Immobilon™ Western Chemiluminescent HRP substrate (Millipore), and the signals were analysed using a Gel Documentation and Image Analysis System (Sage Creation).

### Biological activity assay for chIL-7

The chIL-7 biological activity was evaluated using mouse pre-B cell line (2E8), an IL-7-dependent proliferation cell line [35] as described previously [15].

### Chicken immunization

Two hundred and forty SPF chickens (21–23 day old) were randomly divided into 5 groups of 48 birds each (Table 1). Each group was further subdivided into three sub-groups, for antibody tracing (8 birds), cellular immunity evaluation (8 birds) and challenge (32 birds). The chickens in group 1 remained unimmunized as a negative control. The chickens in groups 2–5 were co-administrated intramuscularly with inactivated IBDV vaccine (10^5^ ELD_50_/0.2, 0.25 mL) plus varying amounts of recombinant chIL-7 (0, 50, 100, 200 μg), and boosted with the same titers of inactivated IBDV vaccine and the same doses of chIL-7 twice at 1-week intervals.

**Table 1:** **Groups of chickens and dosages of recombinant chIL-7 and inactivated IBDV vaccine used in immunization**

Groups	Number of birds	chIL-7 doses (µg)	Inactivated IBDV vaccine (10^5^ ELD_50_/0.2 mL) (mL)
1	48	–	–
2	48	0	0.25
3	48	50	0.25
4	48	100	0.25
5	48	200	0.25

Blood samples were collected by the wing vein in the antibody tracing subgroups (8 birds each) 2 days before immunization and then at 0, 14, 28, 42 and 56 days after the first immunization. Titers of specific antibodies against IBDV were determined by ELISA.

At 30 days post-immunization, the chickens in the cellular immunity evaluation subgroups (8 birds each) were euthanized. Splenic lymphocytes were aseptically prepared by Ficoll density gradient centrifugation for proliferation index measurements by MTT (3-[4,5-dimethylthiazol-2-yl]-2,5-diphenyl tetrazolium bromide, Sigma) colorimetric assay, and IFN-γ and IL-4 productions by ELISA.

At 30 days post-immunization, the chickens in the infection subgroups (32 birds each) were orally infected with the virulent IBDV (Harbin-1 strain) (1 × 10^3^ ELD_50_/each bird). The mortality index, bursal/body ratios (B/B ratios), bursal lesion scores and protection efficiency were evaluated as described below.

### Antibody titers and neutralization titers to IBDV

Antibody titers were measured by ELISA as previously described [15, 16] using the recombinant IBDV VP2 protein as antigen and HRP-conjugated goat anti-chicken IgG (Sigma) as a secondary Ab, and virus neutralization (VN) assays were performed as described previously [15, 16].

### Lymphocyte proliferation assay

Lymphocyte proliferation was assessed by the MTT method [36]. The immunized chickens were euthanized on day 30 and their spleens were removed aseptically. Splenic lymphocytes were separated by density gradient centrifugation on Ficoll-Paque and washed twice in fresh RPMI 1640 medium (Invitrogen). The cells were resuspended at 2 × 10^6^ cells/mL in RPMI 1640 medium with 10% fetal bovine serum (FBS), 100 units/mL penicillin, 100 μg/mL streptomycin, and 2 mM l-glutamine, 100 mL cells were added to 96-well plates and stimulated in vitro for 72 h at 37 °C in a 5% CO_2_ incubator with either concanavalin A (Con A, 5 μg/mL, Sigma) as a positive control, or recombinant VP2 protein (5 μg/mL) as specific antigen. An untreated culture served as a negative control. Twenty microliter MTT (5 mg/mL) were then added to each well and incubation was continued for 4 h. The cells were collected by centrifugation and incubated with 150 μL of dimethylsulfoxide (DMSO) to solubilize intracellular MTT. Supernatants were then transferred to another 96-well plate and *OD*_490_ values were read in a microplate reader.

### Cytokine production

Isolated splenic lymphocytes were stimulated with VP2 protein as above. Chicken IL-4 and IFN-γ concentrations in the culture medium were measured by sandwich ELISA using commercially available chicken ELISA kits (Elabscience) following the manufacturer’s instructions.

### Viral challenge study

At 30 days post-immunization, the chickens in the challenge subgroups (32 birds each) were orally challenged with 1 × 10^3^ ELD_50_ virulent IBDV (propagated in chicken embryos). The challenged chickens were observed clinically for 8 days and mortalities were recorded. Chickens and bursae were weighed and B/B ratios were calculated by (bursal weight/body weight) × 1000. Bursal lesion scores were evaluated based on the histopathological severity of bursae [37]. Protection was defined by the number of chickens with histopathological BF lesion scores of 0 or 1 divided by the number of chickens in the group [37].

### IBDV titulation in bursal tissues and nasal secretions

IBDV tituations was evaluated with TICD_50_ method using DF-1 cells [15]. Briefly, the bursal tissue (200 mg) was homogenized in 800 μL DMEM serum-free medium with small-sized glass homogenizer. After centrifugation, the supernatant was gradually diluted tenfold with the DMEM and then infected DF-1 cells cultured in 96-well plates at 70–80% confluence. The cytopathic effects of infected DF-1 cells were observed at 96 h post-infection. The IBDV titers were determined by the Reed–Muench method and expressed as the 50% tissue culture infective dose (TCID_50_)/g tissue. For IBDV tituations in nasal secretions, the nasal secretions were collected with sterile cotton sticks and washed from the sticks with 500 μL DMEM serum-free medium after weighting (1.005 mg of nasal secretion is proximately equal to 1 μL of nasal secretion). After centrifugation, the supernatant was gradually diluted tenfold with the DMEM, then infected DF-1 cells, the IBDV titers were measured as described above and expressed as TCID_50_/mL secretion.

### Statistics

The significance of differences between groups was evaluated by one-way analysis of variance (ANOVA) with the Dunnett’s post-comparison test for multiple groups to control group, or by the Student’s *t* test for two groups.

## Results

### Successful construction of chicken IL-7 prokaryotic expression vector

**Figure 1:**
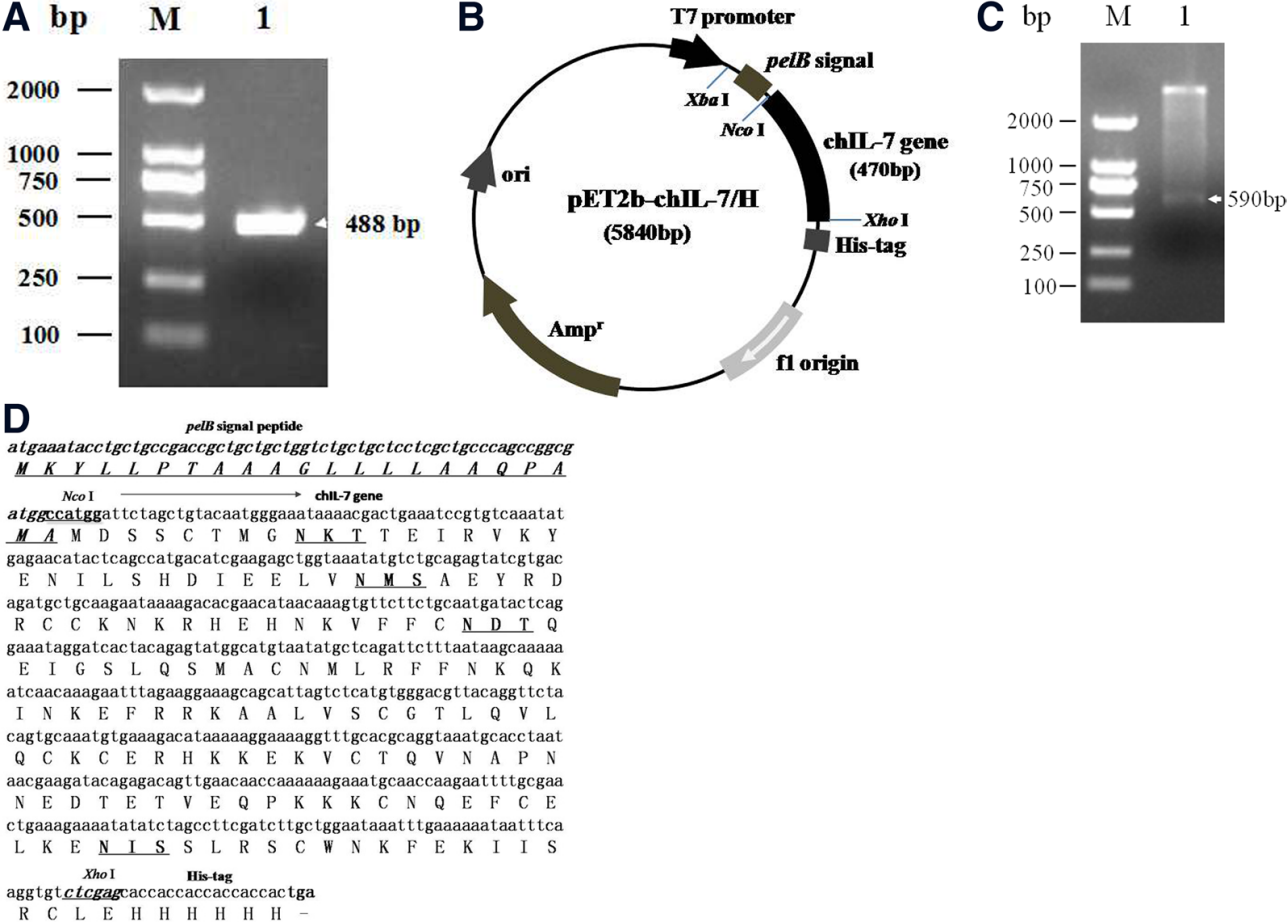
**Construction of chIL-7 prokaryotic expression vector. A** PCR product of chIL-7 gene amplified from pcDNA-chIL-7/MH plasmid. Lane 1, negative control; lane 2, chIL-7 product; M, DL-2000 DNA markers. **B** Structure of pET20b-chIL-7/H vector. The chIL-7 gene fused with *pelB* signal peptide at 5′-terminus and His-tag at 3′-terminus was controlled by T7 promoter. **C** Restriction digestion identification. M, DL-2000 DNA markers; lane 1, pET20b-chIL-7/H plasmid; lane 2, pET20b-chIL-7/H plasmid was digested by *Xba*I and *Xho*I. **D** The chIL-7 gene sequence and the insertion sites in the expression vector. The signal peptide-deleted chIL-7 gene was inserted into pET20b(+) by *Nco*I and *Xho*I sites, fused with *pelB* signal peptide and His-tag. There are 4 potential *N*-glycosylated sites (NXT/S) in chIL-7.

To construct chIL-7 prokaryotic secretory expression vector, the chIL-7 gene without signal peptide was amplified by PCR (Figure 1A) from a chIL-7-containing plasmid, and then sub-cloned into pET20b(+) plasmid to generate chIL-7 prokaryotic secretory expression vector pET20b-chIL-7/H (Figure 1B). The chIL-7 gene sequence and its expression vector structure were confirmed by restriction digestion (Figure 1C) and sequencing (Figure 1D). The results show that the signal peptide-free chIL-7 gene was fused with the prokaryotic signal peptide (*pelB* signal peptide) at its 5′-terminus, and His-tag at its 3′-terminus (Figure 1D).

### Secretory expression in *E. coli* and biological identification of recombinant chIL-7

**Figure 2:**
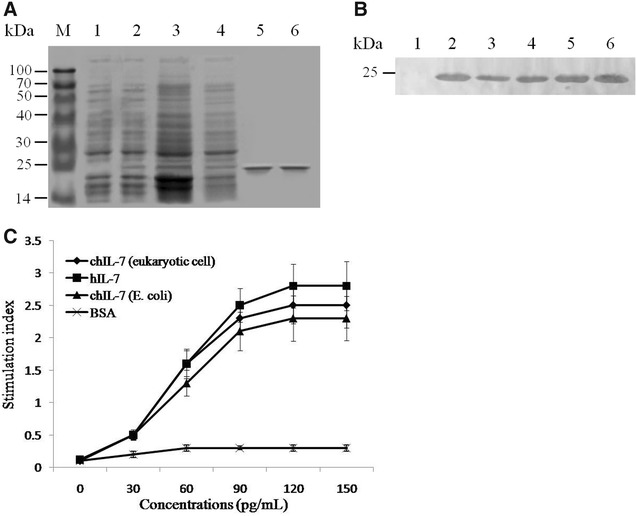
**Identification of chIL-7 expression and its biological activity. A** Identification of chIL-7 protein in different fractions by 10% SDS-PAGE and Coomassie blue staining. M, protein markers; lane 1, whole cell fraction from empty vector-transformed bacteria; lane 2, whole cell fraction from pET20b-chIL-7/H vector-transformed bacteria; lane 3, insoluble fraction; lane 4, cytoplasmic fraction; lane 5, periplasmic fraction; lane 6, purified chIL-7 from periplasmic fraction with Ni–NTA agarose beads. **B** Identification of chIL-7 protein in different fractions detected by Western blot. Lanes 1–6, the samples corresponding to **A**. **C** Identification of chIL-7 biological activity based on its ability to stimulate proliferation of 2E8 cells measured by the MTT assay.

To confirm whether the constructed vector can mediate chIL-7 expression in a secretory manner in *E. coli* and whether large-scale preparation of recombinant chIL-7 can be achieved, BL21 *E. coli* was transformed with pET20b-chIL-7/H plasmid. The transformants were then screened and their expression levels were compared. SDS-PAGE (Figure 2A) and Western blot (Figure 2B) results show that the chIL-7 protein could be detected in all tested fractions (including whole cell, insoluble, cytoplasmic and periplasmic fractions). The highest levels of chIL-7 protein were detected in periplasmic fractions, indicating that the constructed vector could mediate chIL-7 expression in *E. coli* in a secretory manner. In addition, by optimizing expression conditions (under 1 mM IPTG induction at 30 °C for 6 h), high-level expression was achieved, with a yield of up to 50 mg/L fermentation media. Following incubation with mouse immature B lymphocytes (2E8 cells), an IL-7-dependent cell line, measurement of B cell proliferation by the MTT method shows that, like recombinant human IL-7 (Thermo Fisher Scientific) and eukaryotic cell-expressed chIL-7 [30], chIL-7 prepared in *E. coli* had the ability to stimulate 2E8 cell proliferation (Figure 2C).

### Recombinant chIL-7 enhances inactivated IBDV vaccine-induced humoral immune response

**Figure 3:**
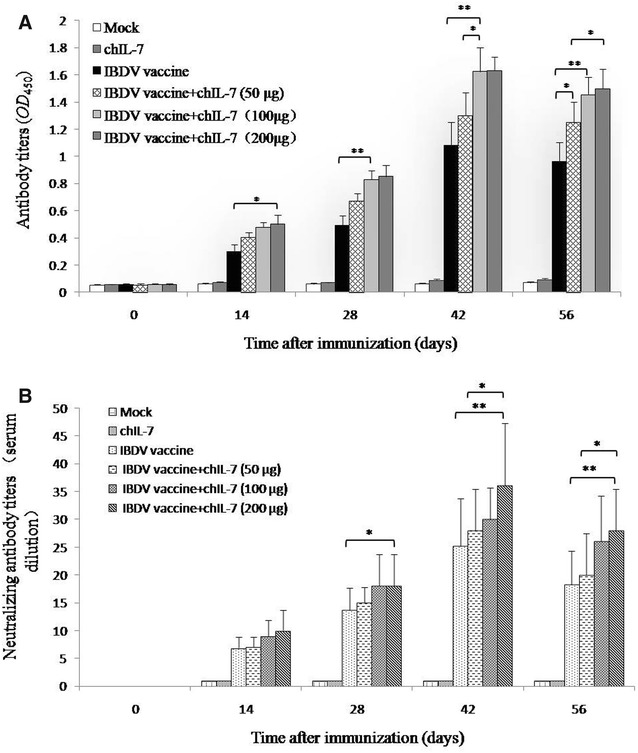
**Titers of antibodies against IBDV in the sera of chickens co-administrated with the inactivated IBDV vaccine and chIL-7 at the different time. A** IBDV antibody titers measured by ELISA over time post-immunization. **B** IBDV neutralizing antibody titers (presented by serum dilution) in the immunized chickens measured by virus neutralization test. Values are expressed as mean ± SD. *P < 0.05; **P < 0.01.

To determine if recombinant chIL-7 had the ability to enhance the chicken humoral immune response to inactivated IBDV vaccine, the SPF chickens were inoculated with inactivated IBDV vaccine and different doses of recombinant chIL-7 (Table 1), and sera were collected at time intervals thereafter. The results show that chIL-7 significantly enhanced antibody titers (Figure 3A). High titers of neutralizing antibodies against IBDV were also detected in the chickens given IBDV and chIL-7 (Figure 3B). These results indicate that recombinant chIL-7 possesses the ability to enhance IBDV vaccine-induced humoral immune response against IBDV. Both VP2 antibody titers and neutralizing anitbody titers reached the highest levels at 42 days post-immunization, but significantly decreased at 56 days post-immunization with IBDV vaccine.

### Recombinant chIL-7 promotes inactivated IBDV vaccine-induced cellular immune response

**Figure 4:**
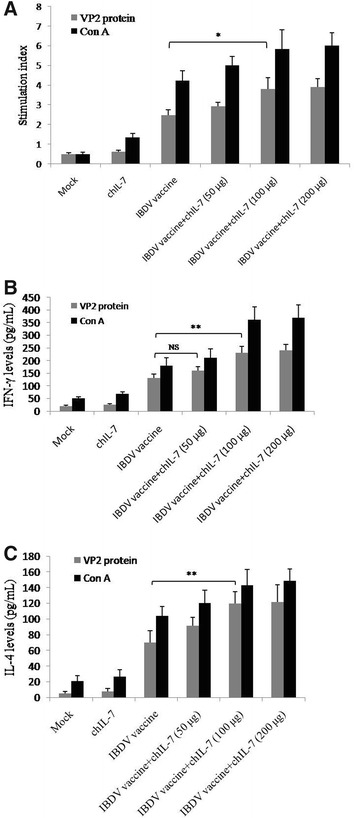
**Measurements of lymphocyte proliferation and cytokine production of co-administrated chickens with inactivated IBDV vaccine and chIL-7. A** Lymphocyte proliferation; **B**, **C** IFN-γ and IL-4 production measured by ELISA. Data are presented as mean ± SD. *P < 0.05; **P < 0.01.

To investigate whether the recombinant chIL-7 can enhance inactivated IBDV vaccine-induced chicken cellular immune responses, an analysis was made of lymphocyte proliferation in chickens treated with inactivated IBDV vaccine/chIL-7 following VP2 protein and Con A stimulation in vitro. The results show that lymphocyte stimulation indices in vaccine/chIL-7 treated chickens were significantly higher than in chickens given inactivated IBDV vaccine alone (*P* < 0.01) at 30 days post-immunization (Figure 4A). To further investigate the effect of chIL-7 on inactivated IBDV vaccine-induced cellular immune responses, IFN-γ and IL-4 production in splenic lymphocytes from immunized chickens was also analyzed by ELISA following stimulation with VP2 protein or Con A in vitro. The results show that the chIL-7 significantly enhanced IFN-γ expression in inactivated IBDV vaccine-immunized chickens (Figure 4B). However, the chIL-7 did not show the same potency in enhancing IL-4 production (Figure 4C), indicating that chIL-7 tends to enhance an inactivated IBDV vaccine-induced Th1 T cell response.

### Recombinant chIL-7 increased the protection of inactivated IBDV vaccine-immunized chickens against virulent IBDV challenge

**Figure 5:**
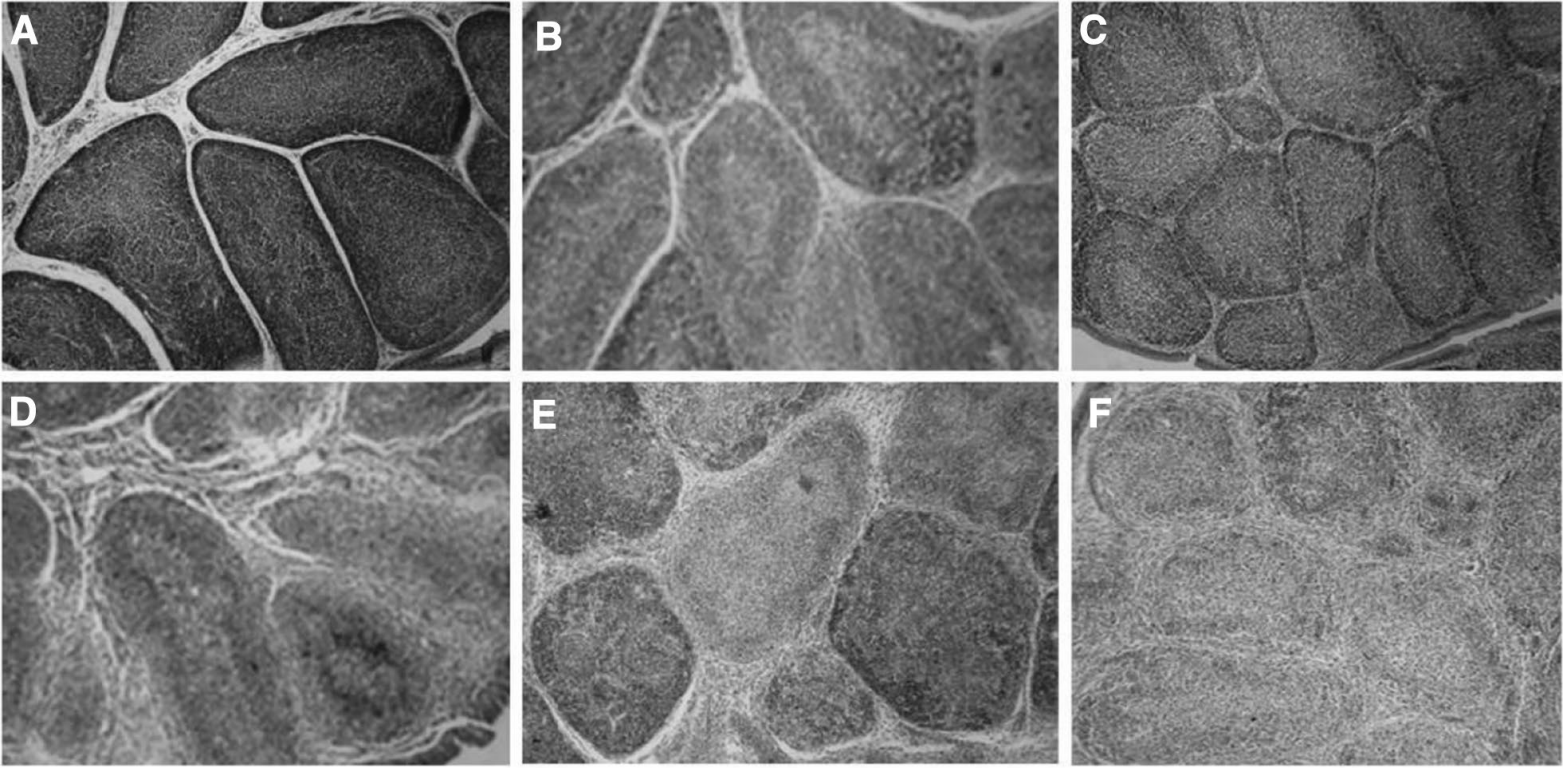
**Bursal lesion score criteria based on bursal histopathological characteristics. A**–**F** Present bursal lesion scores of 0–5, respectively.

To evaluate the enhancement of chIL-7 on the protection conferred by inactivated IBDV vaccine, chickens in the challenge subgroups were infected orally with virulent IBDV at 30 days post immunization. Clinical signs were observed and mortalities were recorded after 5 days. B/B ratios, bursal lesion scores based on bursal histopathological characteristics (Figure 5), protection and IBDV titers in bursal tissues and nasal secretions are presented in Table 2. During the experimental period, chickens in the unchallenged control group remained healthy and had normal sizes of bursae (0 score in Figure 5). The unimmunized (mock) and chIL-7-alone groups showed typical clinical symptoms, with 0 and 6% survival rates, respectively. However, 78–97% of the chickens survived in immunized with inactivated IBDV vaccine alone or inactivated IBDV vaccine plus chIL-7. Importantly, chickens given chIL-7 (100 or 200 µg/mL) along with the inactivated IBDV vaccine displayed higher survival rates (91–97%) than those given inactivated IBDV vaccine alone (78%). Furthermore, chickens given both IBDV vaccine and chIL-7 had higher B/B ratios, lower bursal lesion scores, lower IBDV titers in bursal tissues and nasal secretions than chickens immunized with inactivated IBDV vaccine alone. Protection with vaccine/chIL-7 was 78–94% based on bursal lesion scores (Figure 5), significantly higher (*P* < 0.05) than with vaccine alone (72%). All results indicate that chIL-7 increased the survival rates and protection efficiency of the chickens immunized with inactivated IBDV vaccine.

**Table 2:** **Enhancement by chIL-7 of IBDV vaccine-induced protection following challenge with virulent IBDV**

Groups	Mortality^a^	Survival rate (%)^b^	B/B ratios^c^	Histopathological BF lesion scores^d^						Protection^e^ (%)	IBDV in BT^f^ (TCID_50_)/g	IBDV in NS^g^ (TCID_50_)/mL
				0	1	2	3	4	5			
Control unchallenged	0/32	100	7.31 ± 0.343	32	0	0	0	0	0	N/A	–	–
Mock	32/32	0	1.32 ± 0.277	0	0	0	2	8	22	0	7.87 × 10^8^	4.66 × 10^4^
chIL-7	30/32	6	2.47 ± 0.438	0	0	0	7	7	18	0	2.54 × 10^8^	0.87 × 10^4^
IBDV vaccine	7/32	78	5.85 ± 0.510	18	5	5	2	2	0	72	5.67 × 10^3^	0.72 × 10^2^
IBDV vaccine + chIL-7 (50 μg)	6/32	81	6.21 ± 0.253	20	5	4	2	1	0	78	0.55 × 10^3^	0.07 × 10^2^
IBDV vaccine + chIL-7 (100 μg)	3/32	91	6.52 ± 0.448	23	5	2	2	0	0	88	0.63 × 10^2^	–
IBDV vaccine + chIL-7 (200 μg)	1/32	97	7.12 ± 0.436	27	3	2	0	0	0	94	0.16 × 10^2^	–

^a^Mortality was recorded during a 5-day-period after virus challenge and presented as number of dead/total number of chickens in each group.

^b^Survival rate is defined as the number of chickens surviving viral challenge/the number of chickens in the group.

^c^B/B ratio is calculated by (bursal weight/body weight) × 1000 and presented as the mean ± SD from each group.

^d^Bursal gross lesions are scored from 0 to 5 based on the severity of bursal involvement at time of euthanasia (0: no lesion; 1: slight change, 2: scattered or partial follicle damage, 3: 50% or less follicle damage, 4: 51–75% follicle damage and 5: 76–100% follicle damage).

^e^Protection is defined by the number of chickens with a histopathological BF lesion score of 0 and 1/the number of chickens in the group.

^f^IBDV titers in the bursal tissues (BT) of the immunized chickens measured by the 50% tissue culture infective dose (TCID_50_) method in DF-1 cells.

^g^IBDV titers in the nasal secretions (NS) of the immunized chickens measured by TCID_50_ method in DF-1 cells.

## Discussion

In this study, we prepared a recombinant chIL-7 using the *E. coli* expression system and administered it to chickens together with inactivated IBDV vaccine to evaluate its adjuvant activity. Our data demonstrate that the recombinant chIL-7 possesses potent immunological adjuvant activity, not only enhancing inactivated IBDV vaccine-induced humoral and cellular immune responses, but also increasing IBDV protection against virulent IBDV challenge.

Mammalian IL-7 are glycosylated proteins, containing 2–4 *N*-glycosylated sites. Regulation of T-cell mediated autoimmunity by IL-7 has been reported to be inversely proportional to the extent of its glycosylation [38]. However, considerable research has shown that non-glycosylated recombinant mammalian IL-7 prepared from the *E. coli* expression system is also able to stimulate B- and T-cell development, proliferation and homeostatic regulation [39–41]. Our previous work showed that recombinant chIL-7 prepared from HEK293T cells as a glycosylated protein, could stimulate B cell proliferation, but until the present work it had not been ascertained that non-glycosylated chIL-7 prepared in *E. coli* possessed stimulating activity.

Two forms (soluble and insoluble) of recombinant proteins exist within the *E. coli* host [42]. The latter are frequently associated with inclusion bodies, while the soluble proteins exist either in the cytoplasm or in the periplasm from which they are secreted via their signal peptide sequences. Purification of recombinant proteins from inclusion bodies is a cumbersome, multistep process involving cell disruption, protein isolation and consecutive cycles of denaturation and renaturation for correct protein folding [43]. This is not only time-consuming and costly but retention of the biological activity of the proteins after purification is not easily achieved. In contrast, secretory expression results in naturally folded, biologically active proteins with a high rate of recovery. The purification process of the proteins from the periplasm is therefore much simpler and less costly than the process involving inclusion bodies. Additionally, periplasmic proteins are less likely to incur proteolytic degradation in the host cells. In this study we achieved chIL-7 secretory expression in *E. coli* BL21(DE3) host by using the pET20b(+) prokaryotic vector containing the pelB signal peptide sequence. Additionally, the pET20b(+) vector also contains 6× His-tag sequence downstream of multiple cloning sites for fusing heterologous proteins with the tag, which facilitated protein purification by means of nickel affinity chromatography and identification by Western blot using anti-His antibody.

To explore the mechanism of chIL-7 adjuvant activity, we previously analyzed the effects of chIl-7 on the common receptor γ chain (γ_c_) expression and found that chIL-7 significantly increased γ_c_ expression in chicken lymphocytes [15, 16]. The γ_c_ is a common receptor subunit shared by a common-cytokine-receptor γ_c_ family, including IL-2, IL-4, IL-7, IL-9 and IL-15. All cytokines in this family are immunostimulatory, therefore, promotion of γ_c_ expression suggests an increase in these cytokine functions, which might be related to chIL-7 enhancement on the immunogenicity of IBDV VP2 DNA vaccine. Our previous work on the IL-7 gene as an OVA DNA vaccine adjuvant in a mouse model showed that mouse IL-7 could increase mouse IL-2 receptor α chain expression [44] in lymphocytes, suggesting that IL-7 can increase IL-2 function, which might also contribute to IL-7 enhancement of IBDV vaccine immunogenicity.

Due to its potent enhancement on immunogenicity and protection of inactivated IBDV vaccine, the recombinant chIL-7 prepared in the *E. coli* expression system will be a candidate adjuvant for further clinical investigations.

## Abbreviations

ChIL-7: chiken interleukin-7
Con A: concanavalin A
DMEM: Dulbecco’s Modified Eagle’s medium
DMSO: Dimethyl Sulfoxide
ELD_50_: 50% embryo lethal doses
ELISA: enzyme-linked immunosorbent assay
FBS: fetal bovine serum
HRP: horseradish peroxidase
IBD: infectious bursal disease
IBDV: infectious bursal disease virus
IFN-γ: interferon-γ
IL-4: interleukin-4
IPTG: isopropyl β-D-1-thiogalactopyranoside
MTT: 3-[4,5-methylthiazol-2-yl]-2,5-diphenyl-tetrazolium bromide
OVA: avalbumin
RPMI: Roswell Park Memorial Institute medium
SDS-PAGE: sodium dodecyl-sulfate polyacrylamide gel electrophoresis
ATCC: America Type Culture Collection
TICD_50_: 50% tissue infective culture doses
